# Kinesin-14 HSET may not oppose kinesin-5 Eg5 activity in RPE-1 cells

**DOI:** 10.17912/micropub.biology.000623

**Published:** 2022-08-06

**Authors:** Frederique Carlier-Grynkorn, Daniele Fachinetti, Phong T. Tran

**Affiliations:** 1 Institut Curie, PSL Université, Sorbonne Université, CNRS UMR144, Paris, France; 2 University of Pennsylvania, Department of Cell and Developmental Biology, Philadelphia, PA, United States

## Abstract

Human retinal pigment epithelium RPE-1 cells are immortalized diploid wild-type cells. RPE-1 is increasingly used for studies of spindle assembly dynamics and chromosome segregation. Here, we imaged living RPE-1 cells using the spinning disk confocal microscope and report their complete spindle assembly dynamic parameters. Live-cell experiments enabled ascribing precise timing of function of the kinesin-5 Eg5 and kinesin-14 HSET throughout different phases of mitosis. Eg5 functions at prophase and metaphase, to assemble and maintain spindle bipolarity, respectively. Eg5 inhibition results in spindle collapse during prophase and metaphase, resulting in monoastral/monopolar spindles. HSET functions throughout mitosis to maintain spindle length. HSET degradation results in shorter spindles through all phases of mitosis. Double-inhibition of Eg5 and HSET produces only monoastral/monopolar spindles, indicating that Eg5 and HSET may not be antagonistic in wild-type RPE-1 cells, contrary to previous studies using cancer cells. In the context of spindle assembly, our results highlight potential important differences between RPE-1 and other cancer-derived cell lines.

**Figure 1. The function of kinesin-5 and kinesin-14 in human wild-type RPE-1 cells:**
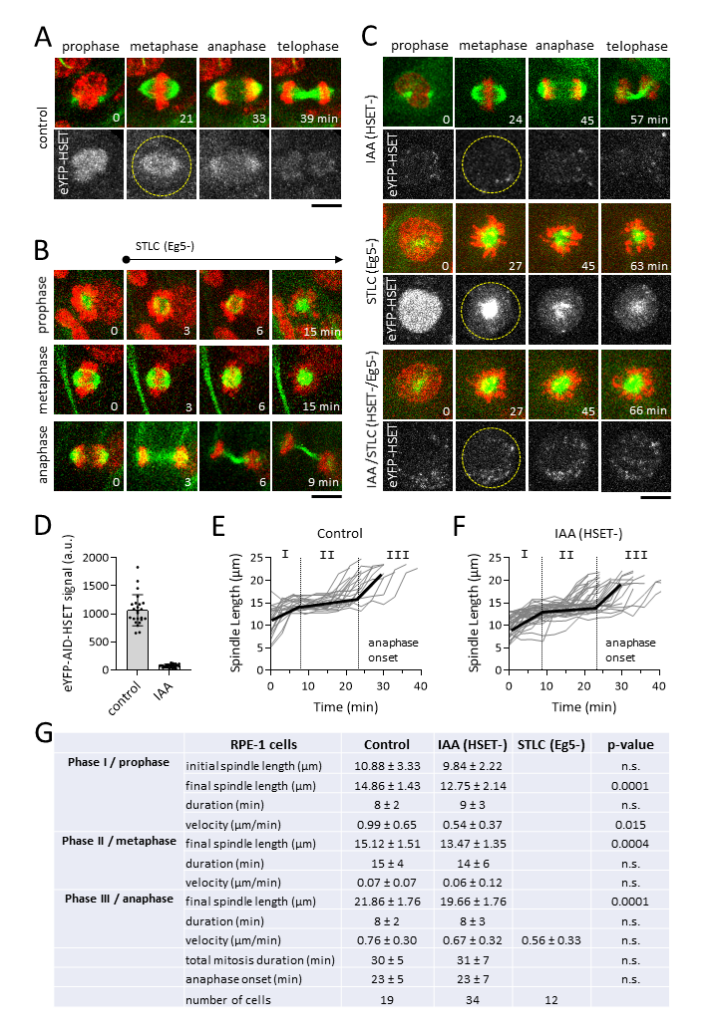
**A.** Time-lapse images of a human RPE-1 cell undergoing mitosis. Color panel (top) shows merged images of microtubules (green) and DNA (red); black-white panel (bottom) shows images of eYFP-HSET signal. Dashed circle outlines the metaphase cell. Scale bar, 10 μm. Kinesin-14 HSET localizes to the nucleus and spindle throughout mitosis (cell is outlined with dashed circle). Scale bar, 10 μm. **B.** Live-cell perfusion of 10 µM STLC, a potent inhibitor of kinesin-5 Eg5. Perfusion of STLC was performed during either prophase (N=11 cells), metaphase (N=13 cells), or anaphase (N=6 cells). Scale bar, 10 μm. **C.** Time-lapse images of mitotic RPE-1 cells treated with auxin to degrade kinesin-14 (HSET-), STLC to inhibit kinesin-5 (Eg5-), and double degradation/inhibition (HSET-/Eg5-). Color panel (top) shows merged images of microtubules (green) and DNA (red); black-white panel (bottom) shows images of eYFP-HSET signal. Dashed circle outlines the metaphase cell. Scale bar, 10 μm. **D.** Plot of eYFP-HSET signal intensity, in arbitrary unit (a.u.), in control and auxin-induced (IAA) HSET degradation in metaphase spindles. Control 1066 ± 276 a.u. (N = 24), IAA 73 ± 276 a.u. (N = 25); HSET was degraded to 7% compared to control. **E.** & **F.** Plot of spindle length vs. time for control (N = 19) and HSET- (N = 36) RPE-1 cells. Spindle elongation showed three distinct phases, representing: I = prophase, II = metaphase, and III = anaphase. Bold black lines show approximate mean spindle dynamics. **G.** Table listing spindle parameters for RPE-1 cells in control, HSET-, and Eg5- cells (mean ± standard deviation, p-value).

## Description

Human hTERT-RPE-1 cells were immortalized, via telomerase expression, from normal somatic retinal pigment epithelium cells ( Jiang et al., 1999 ). These cells are considered normal diploid cells, as they have the proper number of chromosomes and do not exhibit transformed phenotypes. As this cell line has gained prominence as a model system for research on spindle assembly and chromosome segregation, we sought to define its complete spindle assembly parameters throughout mitosis, as well as test the function of kinesin-5 and kinesin-14, two important motors involved in spindle assembly previously reported in cancer cells. Using CRISPR/Cas9 gene-editing, we created an RPE-1 cell line expressing at its genomic loci both alleles of auxin-inducible degron-degradable kinesin-14 eYFP-AID-HSET. We then performed confocal live-cell imaging to visualize HSET, as well as using the vital dye SiR-Tubulin and Hoechst to visualize the microtubules and DNA, respectively. Live-cell imaging enabled tracking mitosis from start to finish, with clear visualization of prophase, metaphase, anaphase and telophase spindles (Fig. 1A). The movies enabled the measurement of spindle length over time, where we observed three distinct spindle elongation phases, correlating with phase I/prophase, phase II/metaphase, and phase III/anaphase (Fig. 1E). The complete spindle elongation parameters for RPE-1 cells are reported (Fig. 1G).

Kinesin-5 Eg5 is essential for bipolar spindle assembly, as the long-term inhibition of Eg5 by S-trityl-L-cysteine (STLC), which inhibits motor binding to microtubules (Chen et al., 2017), creates monoastral/monopolar spindles and subsequent cell death (Ogo et al., 2007). Live-cell imaging enabled precisely-timed introduction of STLC to cells. We observed that STLC-inhibition of Eg5 prevented spindle elongation at prophase, producing monoastral/monpolar spindles (Fig. 1B), indicating that Eg5 is required for bipolar spindle assembly, and consistent with known function of Eg5 (Myers et al., 1999). At metaphase, STLC-inhibition of Eg5 resulted in 78% of spindles collapsing from bipolar back to monoastral/monopolar state (Fig. 1B), consistent with previous reports (Chen et al., 2017; Gayek and Ohi, 2014), and indicating that Eg5 is required to maintain metaphase spindle bipolarity and length. In contrast, STLC-inhibition of Eg5 did not significantly alter anaphase spindle elongation (Fig. 1B), consistent with previous report (Collins et al., 2014; Vukušić et al., 2021), and indicating that Eg5 is not required for anaphase spindle elongation. Indeed, anaphase spindle elongation velocity showed no significant difference between control 0.76 ± 0.30 µm/min versus STLC (Eg5-) 0.56 ± 0.33 µm/min (p=0.12) (Fig. 1G).

Kinesin-14 HSET is essential for cancer cells with supernumery centrosomes, to cluster their centrosomes and prevent multipolar spindles and cancer cell death (Vitre et al., 2020). We tested the function of HSET in RPE-1 cells by degrading HSET via auxin addition. In control cells, HSET localized to the nucleus and prominently on the spindle during mitosis (Fig. 1A). Auxin-induced degron-degradation reduced the signal of HSET on spindles to 7% of control cells, i.e., we achieved 93% HSET degradation (Fig. 1C & 1D). Nevertheless, the overall spindle elongation dynamics did not significantly change compared to control cells (Fig. 1F & 1G). We observed a slight decrease in spindle length throughout mitosis in IAA (HSET-) cells, with a control metaphase length of 15.12 ± 1.51 µm versus IAA (HSET-) 13.47 ± 1.35 µm (Fig. 1G), indicating HSET helps maintain spindle length throughout mitosis, and consistent with a previous report (Cai et al., 2009).

Finally, it has been reported that kinesin-5 and kinesin-14 double-inhibition can restore up to 20-40% spindle bipolarity in cancer cells CFPAC1 (Mountain et al., 1999) and HeLa (Tsui et al., 2009, Watts et al., 2013), indicating that HSET can oppose the activity of Eg5. We tested this in RPE-1 cells by simultaneously degrading HSET and inhibiting Eg5. We observed only monoastral/monopolar spindles in STLC (Eg5-) cells (N=83 cells) (Fig. 1C). Similarly, we observed only monoastral/monopolar spindles in IAA/STLC (HSET-/Eg5-) cells (N=26 cells) (Fig. 1C). In contrast to previous report for some cancer cells, HSET cannot oppose the activity of Eg5 in non-cancer RPE-1 cells. We note that our experiments utilize HSET-degradation via degron and Eg5-inhibition via STLC drug. We speculate that alternative modes of inhibition such as gene-knockout or RNA-interference may produce different results. In summary, our work defines spindle elongation parameters for the different phases of mitosis in the wild-type human RPE-1 cells, and highlights potential important differences between wild-type and cancer cells in their utilization of kinesin motors for spindle assembly dynamics.

## Methods

**Cell culture**

We used human hTERT-RPE-1 (RRID:CVCL_4388) cells ( Bodnar et al., 1998 ), which has been modified into hTERT-RPE-1-TIR1 to include the F-box gene TIR1 that binds the degron (AID) domain upon auxin (IAA) induction (Holland et al., 2012). We introduced an auxin-inducible degron-degradable kinesin-14, eYFP-AID-HSET, into RPE-1-TIR1 at its genomic locus using CRISPR/Cas9 as described (Holland et al., 2012). This cell line was used for all experiments. Cell culture condition is well-established ( Bodnar et al., 1998 ). Briefly, cells were maintained in DMEM/F-12 medium (GIBCO Cat# 21041-025) supplemented with 10% fetal calf serum (Eurobio Cat# CVFSVF-00-01), 1% penicillin-streptomycin (GIBCO Cat# 15140-22) in 5% CO _2_ incubator at 37°C. For Eg5 inhibition, STLC was added to cells 10 min prior to imaging. For degron-induced HSET degradation, cells were treated with IAA 16-20 hr prior to imaging.

**Drugs**

S-trityl-L-cysteine (STLC) (Sigma Cat# 164739) was used at 10 µM concentration.

Indole-3-acetic acid (IAA) (Sigma Cat# I5148) was used at 500 µM concentration.

**Live-cell imaging**

Approximately 16-20 hr prior to imaging, 10 µM Verapamil + 5nM SiR-tubulin (Spirochrome Cat# CY-SC002), and 1 hr prior to imaging, 10 µM Hoechst 33342 (Invitrogen Cat# H3570) dyes were added to the Fluorodish (World Precision Instruments Cat# FD35-100) containing 2 mL of RPE-1 cells. Cells were imaged using the spinning disk confocal microscope. Briefly, the Nikon Eclipse Ti-E perfect focus inverted microscope, with 40X/1.3 NA Plan Apo oil immersion objective lens and Mad City Piezo stepper stage, coupled to the Yokogawa CSU-X1 spinning disk confocal unit, the Photometrics Cascade EM-CCD camera, the Gataca Systems laser unit with Cy5/561 nm (100 mW), GFP/491 nm (100 mW) and UV/405 nm (100 mW) lines, controlled by Molecular Devices software MetaMorph 7.8, enclosed within a thermal box to keep stable temperatures of 37°C. Movies were made with the following parameters: laser power 5%, EM-gain 300, Bin 1X, exposure time 100-200 ms, 13 optical z-sections, 2 µm spacing per 3D stack, 3 min time interval between stacks, and 3 hr movies.

## Reagents

Plasmid to generate eYFP-AID: AddGene #47329

Plasmid to generate eYFP-AID-HSET with Cas9 and single guide RNA background: AddGene #42230

Primers to generate eYFP-AID:

sense : ATGGTGAGCAAGGGCGAGG

antisense : GCTCGAAGCTCTGCTCTT

Primers to generate eYFP-AID-HSET:

500 bp before HSET start:

sense : CCCTTCTCCTCCGGCCG

antisense : GTCCACCTCGGTCCCAC

500 bp after HSET start:

sense : GATCCGCAGGTGAGTAGG

antisense : AGATTACCCTACTCTCCTTC

single RNA-guide sequence:

CTGCATTCCCCCGGCGCGTGTGG

Cell line used :

RPE-1-TIR1 (Holland et al., 2012)

RPE1-1-eYFP-AID-HSET (this study)
